# The impact of EMA recommendations on the real-life use of Janus kinases inhibitors for rheumatoid arthritis: the Expanded Risk Score in RA as a tool to quantify the risk of cardiovascular events

**DOI:** 10.3389/fimmu.2023.1225160

**Published:** 2023-08-31

**Authors:** Ennio Giulio Favalli, Gilberto Cincinelli, Sabino Germinario, Raffaele Di Taranto, Francesco Orsini, Gabriella Maioli, Martina Biggioggero, Matteo Ferrito, Roberto Caporali

**Affiliations:** ^1^ Department of Rheumatology and Medical Sciences, ASST Gaetano Pini-CTO, Milan, Italy; ^2^ Department of Clinical Sciences and Community Health, Università degli Studi di Milano, Milan, Italy

**Keywords:** rheumatoid arthritis, JAK inhibitors, b/tsDMARDs, safety, cardiovascular risk

## Abstract

**Objective:**

To evaluate in patients with rheumatoid arthritis (RA) the impact of EMA recommendations on the real-life prescription of JAK inhibitors (JAKis) and the use of the Expanded Risk Score in RA (ERS-RA) to quantify the risk of major adverse cardiac events (MACE).

**Methods:**

We conducted a retrospective analysis of real-life RA patients treated with JAKis. Patients were classified as ineligible for JAKis if they fulfilled EMA criteria (>65 years-old, history of malignancy, or increased risk of venous thromboembolic events [VTE] or MACE including smoking). Risk of MACE was defined according to ORAL Surveillance trial inclusion criteria (ORALSURV) or by using the ERS-RA.

**Results:**

Of 194 patients enrolled, 57.9% were classified as ineligible according to EMA definition (ORALSURV criteria). The most frequent reason for ineligibility was increased MACE risk (70.2%), followed by age>65 (34.2%), smoking (30.7%), and increased risk of VTE (20.2%) or malignancy (7%). The use of the ERS-RA reduced the rate of patients carrying an increased CV risk to 18.6% (p<0.001 versus ORALSURV), leading to 46.4% overall ineligible patients. Over a drug-exposure of 337 patient/years, we observed 2 VTE, one MACE (non-fatal stroke), and one solid malignancy (all in the group of patients classified as ineligible according to both the definitions).

**Conclusions:**

Rigorous application of EMA indications in clinical practice could result in the exclusion of a large proportion of RA patients from treatment with JAKis. A proper quantification of the risk for MACE by dedicated tools as ERS-RA is advocated to better tailor the management of RA.

## Introduction

Rheumatoid arthritis (RA) is a chronic immune-mediated inflammatory disease that affects approximately 0.4% of the world’s population (1). RA is characterized by a systemic inflammatory process that mainly occurs in the joints, but can also generate extra-articular manifestations such as increased cardiovascular (CV) risk, neoplastic, infectious diseases and thromboembolic complications (2). In the last decade, the therapeutic armamentarium for the treatment of RA has been enriched with the introduction of Janus kinase inhibitors (JAKis) that have joined disease-modifying biologics (bDMARDs) as second-line therapy in methotrexate failures (3). After the marketing of the first JAKi tofacitinib in 2012, three other members of the family (baricitinib, upadacitinib, and filgotinib) were licensed for RA therapy, demonstrating similar or even superior efficacy to bDMARDs such as adalimumab (4).

However, in the randomized controlled trials (RCTs) of tofacitinib and baricitinib, some concerns had arisen about the safety profile of JAKis regarding a possible increased risk of serious infections (5), malignancy, and venous thromboembolic events (VTE) (6, 7), which prompted the US Food and Drug Administration (FDA) to request post-marketing RCTs comparing JAKis with TNF inhibitors (TNFis) in a head-to-head design with a primary safety endpoint. Results from the first of these RCTs (ORAL SURVEILLANCE) suggested a potential increased risk for major adverse CV events (MACEs), malignancy, and opportunistic infections associated with tofacitinib compared to TNFis (8).

Moreover, an increase in thromboembolism incidence emerged from a multi-database study of RA patients from routine care treated with baricitinib compared to TNFis (9)

On the basis of this evidence, in February 2022 the European Medicine Agency (EMA) initiated a procedure to review the entire safety profile of the JAKi class, which recently led to a final pronouncement limiting the prescription of all JAKis in subjects over 65 years of age, current or former long-term smokers, and/or considered to be at increased risk of developing CV, neoplastic or thromboembolic complications (10). These limitations are expected to have a considerable impact on clinical practice, both in terms of new JAKi prescriptions and management of at-risk patients already receiving a JAKi with a good clinical response. In this scenario, the correct definition of at-risk patients to be excluded from therapy will be crucial for the proper use of JAKis in real-life.

Therefore, we conducted a retrospective analysis of our cohort in order to determine the proportion of RA patients treated with JAKis who fall under the EMA’s suggested definition of patients at risk. The aim of the study was also to evaluate in a real-life setting the performance of a validated score for quantifying CV risk in RA (Expanded Risk Score in RA (ERS-RA)) (11) in predicting the occurrence of MACEs in subjects who are candidates for treatment with JAKis.

## Materials and methods

### Patients and study design

The source for the cohort enrolled in this study was a local population-based registry established in 2002 with the aim of collecting demographic and clinical data of all the patients treated with biologic or targeted synthetic DMARDs in a tertiary-care Rheumatology Centre of Northern Italy. The registry was approved by the Gaetano Pini Institute Ethics Committee (approval n. 150_2002) and included all patients who signed the informed consent for any subsequent retrospective analysis of collected data. All analyzed clinical information are reported as anonymous aggregate data, excluding any identifiable medical information.

Within the registry, the current analysis was retrospectively limited to ≥18 years old RA patients (fulfilling the ACR/EULAR 2010 RA classification criteria) who received a treatment with tofacitinib, baricitinib, upadacitinib, or filgotinib since December 2017, until the database lock on 31^st^ December, 2022. Treatments were administered in routine care in accordance with international recommendations: JAKis were prescribed according to licensed regimen and concomitant csDMARDs and/or corticosteroids were administered if ordered by the referring rheumatologist.

### Outcomes and study design

Demographic (gender, age, body mass index (BMI), smoking status), therapeutic (previous and concomitant treatment with csDMARDs and/or corticosteroids), and clinical (disease duration, rheumatoid factor (RF) and/or anti-citrullinated protein antibodies (ACPA) positivity, presence of RA extraarticular manifestations or comorbidities) data were extracted at the time JAKi was started and when the database was closed. In particular, comorbid conditions associated to increased risk of CV disease (arterial hypertension, heart failure, hyperlipidemia, diabetes mellitus, non-ischemic heart disease, history of coronary artery disease (CAD) or stroke), VTE, or malignancy were noted.

Clinimetric indices were collected at baseline and every 3 months to monitor disease activity (by simplified or clinical disease activity index (SDAI/CDAI)) and disability (by Health Assessment Questionnaire Disability Index (HAQ-DI)) over time.

**Figure 1:**
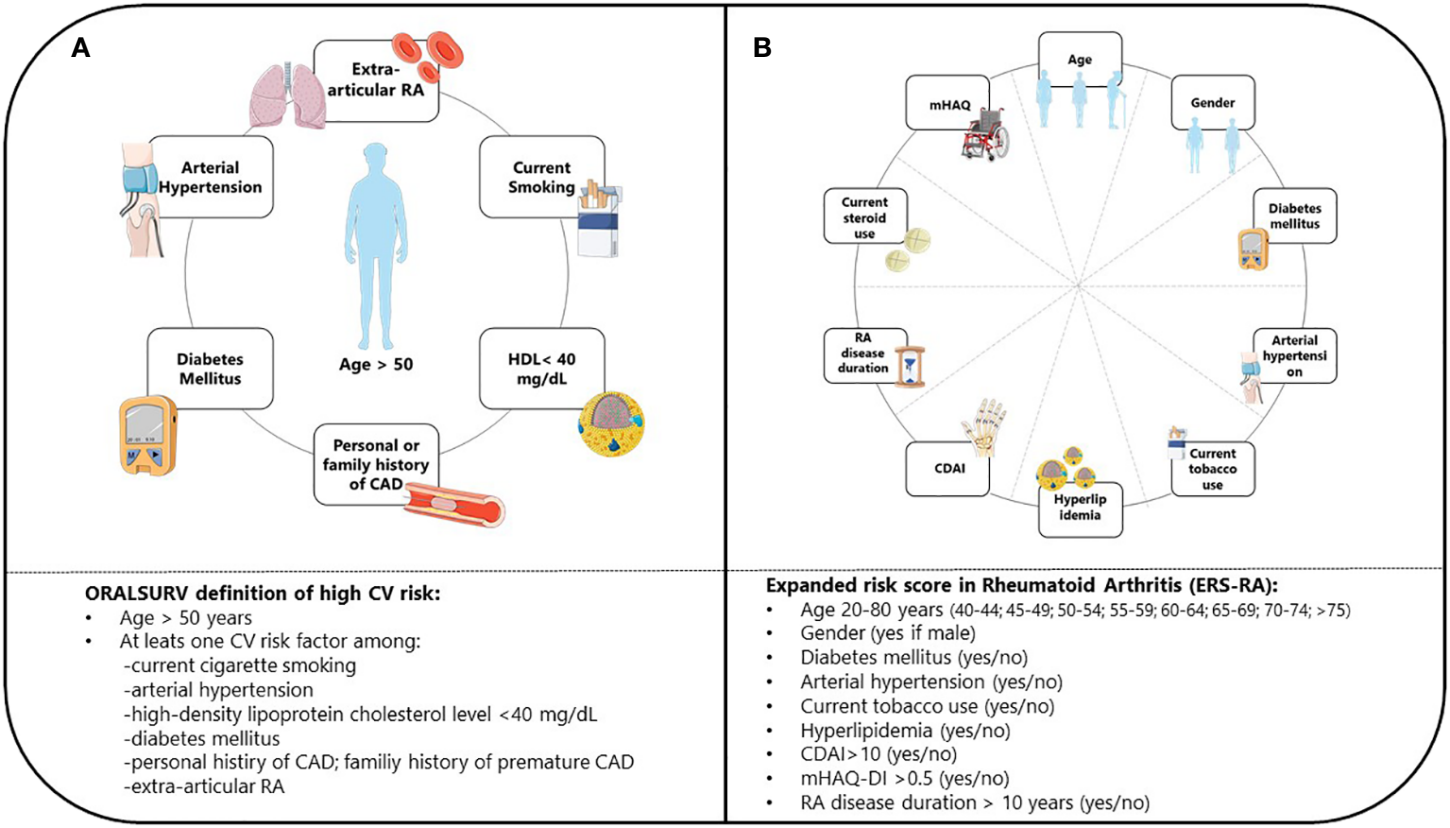
Definition of CV risk according to ORAL SURVEILLANCE trial **(A)** and ERS-RA **(B)** CV, Cardiovascular; ERS-RA, Expanded Risk Score in Rheumatoid Arthitis; HDL, High-Density Lipoprotein; CAD, Coronary Artery Disease; RA, Rheumatoid Arthritis; CDAI, Clinical Disease Activity Index; mHAQ-DI, Modified Health assessment Questionnaire Disability Index.

Patients were classified as ineligible for JAKis according to the EMA’s definition if at baseline they were i) older than 65; ii) current or former long-term smokers; iii) carrying an increased risk of VTE (according to International Society of Thrombosis and Haemostasis categorization (12)), cancer (defined as previous history of malignancy), or MACE. For the initial prevalence analysis (ORALSURV definition), the latter was defined as the inclusion criteria of the ORAL SURVEILLANCE trial (8): being older than 50 and having at least one CV risk factor among i) current cigarette smokers; ii) arterial hypertension; iii) high-density lipoprotein cholesterol level of < 40 mg/dL; iv) diabetes mellitus; v) family history of premature CAD; vii) extra-articular RA; viii) history of CAD (**Figure 1A**). In the subsequent reassessment of risk for MACEs (ESR-RA definition), we used the Expanded Risk Score in RA (ERS-RA), developed and internally validated in 2015 using data from the population of the CORRONA registry (11). The ERS-RA confers a 10-year CV risk and incorporates in the estimation: i) age; ii) gender; iii) diabetes; iv) hyperlipidemia; v) hypertension; vi) current tobacco use; vii) CDAI score; viii) mHAQ score; ix) steroid use; x) RA disease duration. A 10% increase in the 10-year risk was used as the definition of increased CV risk leading to ineligibility to JAKis (**Figure 1B**).

A descriptive analysis of individual reasons for contraindication to JAKis was conducted by using each of the two definitions of increased CV risk. Lastly, an analysis of time to and reasons for JAKi discontinuation was performed in the whole population and in the eligible and ineligible subgroups.

### Statistical analysis

Descriptive results of continuous variables are reported as median or mean values, as appropriate, whereas categorical variables are reported as numbers and percentages. Normality of the data was tested with both Shapiro-Wilk and Kolmogorov-Smirnov tests. Differences in the variable distribution were analysed with the Mann-Whitney U test and chi-squared test for continuous and categorical variables, respectively. A p value ≤ 0.05 was considered statistically significant. Statistical analysis was performed using Stata Statistical Software: Release 17.0 (College Station, TX: StataCorp LLC.).

## Results

### Baseline population characteristics

The study population included 194 RA patients treated with JAKis. Patients’ characteristics are summarized in **Table 1**. The vast majority were female (n=165, 85.1%) and seropositive either for RF and/or ACPA (n=121, 62.4%), with a mean (± standard deviation) age of 55.4 (± 12) years and a mean disease duration of 16.36 (± 10.75) years. Thirty-five (18%) patients were current or former long-term smokers, while 69 (35.6%) were overweight and 21 (10.8%) obese. The median body mass index (BMI) was 23.9 (95% confidence interval (95% CI) 21.3-26.6).

**Table 1:** Baseline characteristics of study population.

	Global population (N=194)
General characteristics	
- **Age, years (mean ± SD)**	55.4 ± 12
- **Male sex, n (%)**	29 (14.9%)
- **BMI, (mean ± SD)**	23.88 (CI 21.36 – 26.59)
- **Overweight (BMI > 25), n (%)**	69 (35.6%)
- **Obese (BMI >30), n (%)**	21 (10.8%)
- **Smokers, n (%)**	35 (18.0%)
Disease characteristics	
- **Disease duration, years (mean ± SD)**	16.31 ± 0.77
- **RF and/or ACPA positive, n (%)**	121 (62.4%)
- **mHAQ-DI, (median [95% CI])**	0,675 [0.125-1.25]
Current therapies	
- **Corticosteroid users, n (%)**	79 (40.7%)
- **MTX users, n (%)**	81 (41.7%)
- **Tofacitinib, n (%)**	56 (28.9%)
- **Baricitinib, n (%)**	89 (45.9%)
- **Upadacitinib, n (%)**	22 (11.3%)
- **Filgotinib, n (%)**	27 (13.9%)
- **First-line targeted therapy, n (%)**	81 (41.7%)
- **1 previous bDMARD, n (%)**	36 (18.6%)
- **>1 previous bDMARD, n (%)**	77 (39.7%)
- **D2T patients, n (%)**	66 (34%)
Comorbidities	
- **Arterial hypertension, n (%)**	59 (30.4%)
- **IHD, n (%)**	3 (1.6%)
- **Non-IHD, n (%)**	18 (9.3%)
- **Heart failure, n (%)**	4 (2.1%)
- **Stroke, n (%)**	2 (1.0%)
- **Thrombosis (VTE/PE), n (%)**	2 (1%)
- **Trombophilia, n (%)**	23 (11.9%)
- **Hyperlipidemia, n (%)**	71 (36.6%)
- **Diabetes mellitus, n (%)**	10 (5.2%)
- **Solid malignancy, n (%)**	8 (4.1%)

BMI: Body Mass Index; RF: Rheumatoid Factor; ACPA: Anti-Citrullinated Protein Antibodies; mHAQ-DI: modified Health Assessment Questionnaire Disability Index; MTX: methotrexate; bDMARDs: biologic Disease-Modifying Anti-Rheumatic Drugs; D2T: Difficult-to-Treat; JAKi: Janus-Kinase Inhibitor; IHD: Ischemic heart disease: MACE: Major Adverse Cardiovascular Events; VTE: Venous Thromboembolism; PE: Pulmonary Embolism.

As to CV comorbidities, 59 (30.4%) of patients were suffering from arterial hypertension, 18 (9.3%) from non-CAD heart disease, 71 (36.3%) from hyperlipidemia, and 10 (5.2%) from diabetes, while 3 (1.6%) had a history of CAD and 2 (1.0%) of stroke. Twenty-three (11.9%) patients (all not receiving an anticoagulant treatment) carried VTE risk factors but only 2 (1.0%) experienced a previous VTE (both deep vein thrombosis [DVT]). A history of malignancy was observed in 8 (4.1%) patients (all solid cancers), half of them on concomitant methotrexate therapy.

Eighty-nine (45.9%) patients were treated with baricitinib, 56 (28.9%) with tofacitinib, 27 (13.9%) with filgotinib, and 22 (11.3%) with upadacitinib.

JAKi was used as the first targeted therapy after failure of MTX in 75 (38.6%), after failure of a previous bDMARD in 34 (17.5%), or as a subsequent line of therapy in 75 (38.6%) patients. According to EULAR definition (13), 66 (34%) were difficult-to-treat (D2T) patients. No patient experienced a switch from one JAKi to another. Concomitant MTX was prescribed in 82 (43.2%) patients, while 79 (40.7%) also received corticosteroids (mean dose 5 mg/daily).

### Population eligibility to JAKis therapy

**Figure 2:**
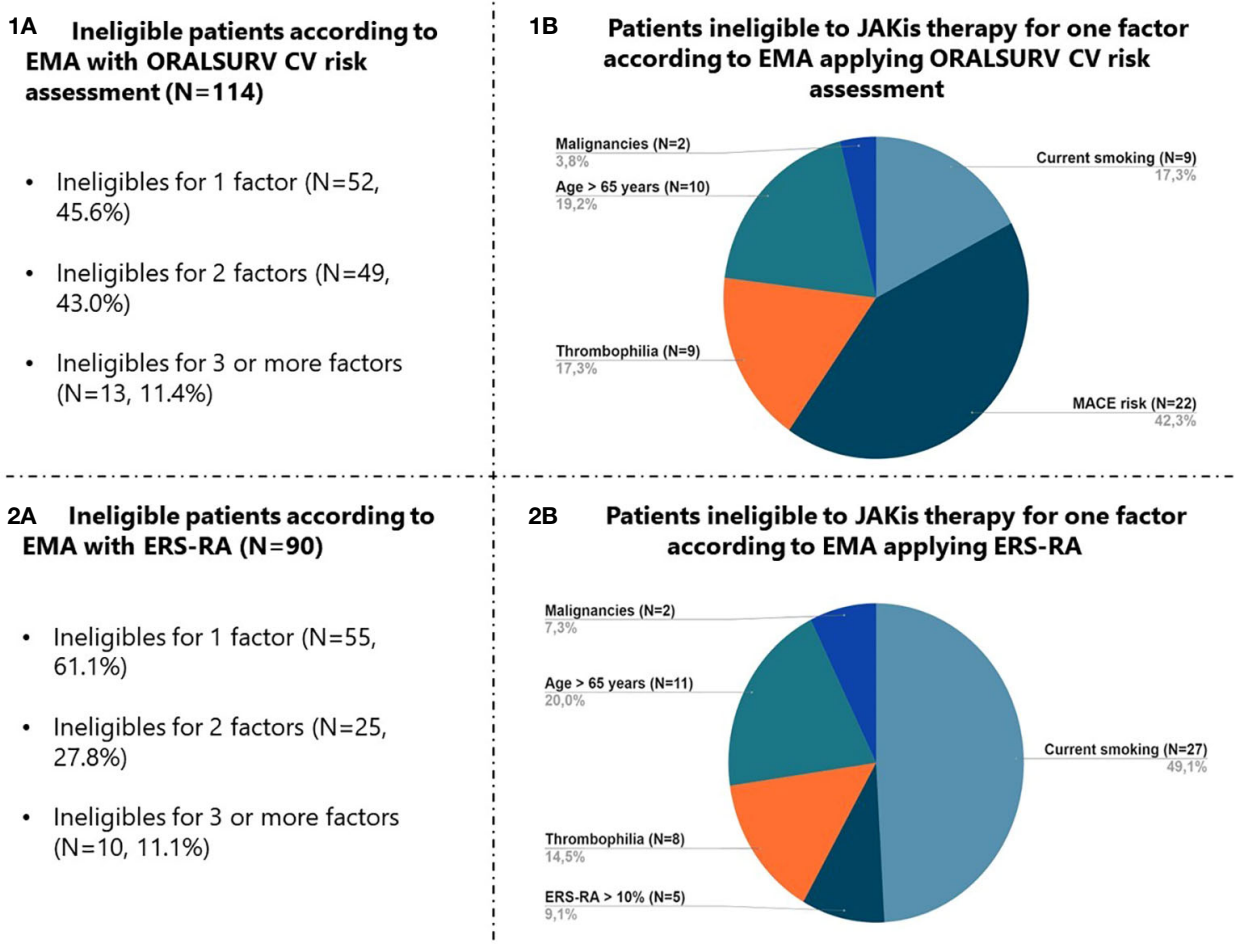
Focus on factors leading to JAKis therapy ineligibility according to EMA with application of ORALSURV CV risk assessment (1A, 1B) and ERS-RA (2A, 2B). JAKis, Janus Kinase Inhibitors; EMA, European Medicines Agency; ORALSURV, ORAL Surveillance trial; CV, Cardiovascular; ERS-RA, Expanded Risk Score in Rheumatoid Arthritis.

The eligibility of the study population to be treated with JAKis was assessed in accordance with the guidance provided by EMA, initially using the inclusion criteria from the ORAL SURVEILLANCE trial to define increased CV risk (ORALSURV definition). Using this definition, 114 (58.7%) patients were classified as ineligible, of whom 38 (33.3%) were MTX insufficient responders, 76 (66.7%) had previously failed one or more bDMARDs and 44 (38.6%) were D2T. In detail, 80 (70.2%) had an increased risk of MACE, 39 (34.2%) were >65 years old, 35 (30.7%) were current/past smokers, 23 (20.2%) carried an increased risk of VTE, and 8 (7.0%) had a history of malignancy. The majority of patients fell under this definition of ineligibility because of a single factor (n=52, 45.6%) or two factors (n=49, 43.0%), while a minority (n=13, 11.4%) showed three or more factors (**Figure 2** – 1A). Among patients fulfilling only one criterion, 22 (42.3%) had an increased CV risk, 10 (19.2%) were older than 65 years, 9 (17.3%) were smokers, 9 (17.3%) carried risks for VTE, and only 2 (3.8%) had a history of malignancy (**Figure 2** – 1B).

Compared to the eligible group, ineligible patients were more frequently obese (18.4% vs 0%; p<0.001), had higher disease duration (17.88 ± 10.86 vs 14.09 ± 10.27; p=0.015), mHAQ score (0.875, CI 0.125 - 1.625; vs 0.125, CI 0 - 375; p<0.001), a higher prevalence of arterial hypertension (43.0% vs 12.5%; p<0.001), non-ischemic heart disease (13.2% vs 3.8%; p=0.03), hyperlipidemia (53.5% vs 12.5%; p<0.001), and diabetes (7.9% vs 1.3%; p=0.04). Compared to whom in the eligible group, ineligible patients received less frequently JAKis as first line therapy after csDMARDs failure: 38 (33.3%) vs 37 (46.2%), p=0.011. The two groups did not significantly differ in terms of D2T patients: 37 ineligible patients (32.4%) vs 29 eligible patients (36.2%), p=0.583 (**Table 2**).

**Table 2:** Bivariate analysis on patients eligible vs non-eligible for JAKis therapy according to EMA applying ORAL SURVEILLANCE CV risk assessment and applying ERS-RA CV risk score.

	ORAL SURVEILLANCE CV risk assessment			ERS-RA CV risk score		
	EMA non-eligible (N=114)	EMA eligible (N=80)	p-value	EMA non-eligible (N=90)	EMA eligible (N=104)	p-value
General characteristics						
- **Age, years (mean ± SD)**	59.33 ± 12.14	49.97 ± 8.3	**<0.001**	60.97 ± 12.99	50.09 ± 7.98	**<0.001**
- **Male sex, n (%)**	17 (14.9%)	12 (17.8%)	0.99	16 (17.8%)	13 (12.6%)	0.3
- **BMI, (mean ± SD)**	24.39 (CI 22.1 – 28.76)	23.05 (CI 21.0 – 25.3)	**0.004**	24 (CI 22.1 – 28)	23.53 (CI 21.09 – 26)	0.12
- **Overweight (BMI > 25), n (%)**	48 (42.1%)	37 (46.2%)	**0.03**	37 (41.1%)	32 (30.8%)	0.18
- **Obese (BMI >30), n (%)**	21 (18.4%)	0 (0%)	**<0.001**	14 (15.6%)	0 (0%)	**<0.001**
- **Smokers, n (%)**	35 (30.7%))	0 (0%)	**<0.001**	35 (38.9%)	0 (0%)	**<0.001**
Disease characteristics						
- **Disease duration, years (mean ± SD)**	17.88 ± 10.86	14.09 ± 10.27	**0.015**	18.63 ± 11.05	14 ± 9.98	**0.002**
- **RF/ACPA positive, n (%)**	72 (63.1%)	49 (61.25%)	0.81	59 (65.6%)	62 (59.6%)	0.3
- **mHAQ-DI, (median [95% CI])**	1 (0.25 – 1.625)	0.125 (0 – 0.375)	**<0.001**	0.875 (0.375 – 1.5)	0.5 (0.125 – 1)	**0.009**
Current therapies						
- **Corticosteroid users, n (%)**	42 (36.8%)	37 (46.2%)	0.20	39 (43.3%)	40 (38.5%)	0.84
- **MTX users, n (%)**	44 (38.6%)	37 (46.2%)	0.286	38 (42.2%)	43 (41.3%)	0.42
- **First-line targeted therapy, n (%)**	38 (33.3%)	37 (46.2%)	**0.011**	31 (34.4%)	44 (42.3%)	**0.013**
- **D2T patients, n (%)**	37 (32.4%)	29 (36.2%)	0.583	30 (33.3%)	36 (34.6%)	0.36
- **Tofacitinib, n (%)**	33 (28.9%)	23 (28.7%)		27 (30.0%)	29 (27.9%)	
- **Baricitinib, n (%)**	55 (48.3%)	34 (42.5%)		39 (43.3%)	50 (48.1%)	
- **Upadacitinib, n (%)**	12 (10.5%)	8 (10.0%)		14 (15.6%)	8 (7.7%)	
- **Filgotinib, n (%)**	14 (12.3%)	15 (18.8%)		10 (11.1%)	17 (16.3%)	
** JAKi discontinuation**	45 (39.5%)	35 (43.8%)	0.8	36 (40%)	44 (42.3%)	0.82
· **MACE**	1	0		1 (nonfatal stroke)	0	
· **Malignancy**	1	0		1 (solid)	0	
· **Thrombosis (VTE/PE)**	2	0		2 (VTE)	0	
** JAKis therapy duration (months, SD)**	17.5 (9 – 41)	10 (5 – 26)	**0.02**	17 (8 – 35)	14 (5 – 34)	0.42
Comorbidity						
- **Arterial hypertension**	49 (43.0%)	10 (12.5%)	**<0.001**	38 (42.2%)	21 (20.2%)	**0.001**
- **IHD**	2 (%)	1 (%)	0.78	2 (2.2%)	1 (0.9%)	0.48
- **Non-IHD**	15 (%)	3 (%)	**0.03**	14 (15.6%)	4 (3.8%)	**0.005**
- **Heart failure**	4	0	0.09	4 (4.4%)	0 (0%)	**0.03**
- **Stroke**	2	0	0.24	2 (2.2%)	0 (0%)	0.13
- **Thrombosis (VTE/PE)**	2	0	0.24	1 (1.1%)	0 (0%)	0.28
- **Trombophilia**	23	0	**<0.001**	16 (17.7%)	0 (0%)	**<0.001**
- **Hyperlipidemia**	61	10	**<0.001**	42 (46.7%)	29 (27.8%)	**0.007**
- **Diabetes**	9	1	**0.04**	8 (8.8%)	2 (1.9%)	**0.03**
** History of Malignancy**	8	0	**0.016**	8 (8.8%)	0	**0.02**
- **Solid**	8	0		8	0	
- **Hematologic**	0	0		0	0	
- **NMSC**	0	0		0	0	
- **Melanoma**	0	0		0	0	

Body Mass Index; RF: Rheumatoid Factor; ACPA: Anti-Citrullinated Protein Antibodies; mHAQ-DI: modified Health Assessment Questionnaire Disability Index; MTX: methotrexate; bDMARDs: biologic Disease-Modifying Anti-Rheumatic Drugs; D2T: Difficult-to-Treat; JAKi: Janus-Kinase Inhibitor; MACE: Major Adverse Cardiovascular Events; VTE: Venous Thromboembolism; PE: Pulmonary Embolism; IHD: ischaemic heart disease; NMSC: non melanoma skin cancer.

Bold means p statistically significant.

In view of the high proportion of patients classified as ineligible due to an assumed increased CV risk based on the ORALSURV definition (8), we decided to re-quantify this variable using a validated score for patients with RA such as the ERS-RA.

By the application of the ESR-RA, the number of patients at increased CV risk dropped to 36 (18.6%; p<0.001 versus ORALSURV definition), resulting in a decrease in the total number of ineligible patients to 90 (46.4%; p<0.001 versus ORALSURV definition). The majority of the patients were ineligible because of one factor only (n=55, 61.1%) (**Figure 2** – 2A), and in this case being a smoker was the most frequently present criterion (n=27, 49.1%) (**Figure 2** – 2B).

Similar to the previous evaluation, a higher proportion of obese (15.6% vs 0%; p<0.001), a higher mHAQ score (0.875, CI 0.375 - 1.5; vs 0.5, CI 0.125 - 1; p=0.009), and a higher prevalence of arterial hypertension (42.2% vs 20.2%; p=0.001), non-CAD heart disease (15.6% vs 3.8%; p=0.005), hyperlipidemia (46.7% vs 27.8%; p=0.007), and diabetes (8.8% vs 1.9%; p=0.03) were observed in the ineligible subgroup compared with the eligible one (**Table 2**).

### Reasons for JAKi discontinuation

During a mean follow-up period of 20.9 ± 17.2 months (overall exposure 337 patient/years), 80 patients discontinued the treatment with JAKi, mainly because of inefficacy (n=50, 62.5%; 21 primary and 29 secondary non-response). Four patients (5%) stopped the treatment due to poor compliance. Safety/tolerability reasons of withdrawal included intolerance (n=9, 11.3%), non-serious infections (n=5, 6.3%), and laboratory tests abnormalities (n=3, 3.8%; two blood count alterations and one elevation of liver enzymes). Among adverse events of special interest leading to discontinuation, we observed 4 (5%) cases of Herpes Zoster virus reactivation; one cases of MACE (a non-fatal stroke occurred in a 65 years-old, non-smoker, obese woman suffering from hypertension and treated with baricitinib); two cases of DVT (one in a 60 years-old, non-smoker woman; and one in a 76 years-old, non-smoker woman suffering from hypertension and hyperlipidemia, both treated with tofacitinib); and one case of solid malignancy (breast cancer) in a 56 years-old, smoker woman with no additional risk factors treated with baricitinib.

The two cases of DVT and the case of malignancy occurred in patients classified as ineligible for JAKis according to both sets of criteria used. The single case of MACE observed occurred in a patient classified as ineligible for JAKis according to both sets of criteria used.

## Discussion

To our best knowledge, this is the first study focused on analyzing the effects of the recently released recommendations by EMA on the treatment of chronic inflammatory diseases with JAKis. Our data showed that the strict application of the EMA indications would result in the potential exclusion from therapy of a very large segment (up to almost 60%) of the RA patient population treated so far with JAKis. This would have a considerable impact on the current management of RA in clinical practice, where JAKis are widely used, and would eventually severely limit the use of a drug class that has proven to be very effective in many different disease subsets in recent years. This impact would firstly affect patients deemed ineligible who are currently receiving a JAKi with benefit, who would have to switch to another mechanism of action without the certainty of being able to confirm the same clinical efficacy experienced with JAKis. The scenario is made even more complicated by the common use of JAKis in patients who are multi-refractory to 2 or more previous bDMARDs (14) or who meet the EULAR definition of D2T RA (13), which also in our cohort represent 39.7% and 34% of patients, respectively. Since within these subgroups the proportion of ineligible patients was significant (66.2% and 32.4% respectively), discontinuing therapy in these cases raises the issue of a difficult alternative therapeutic choice due to the limited number of additional suitable drugs. In particular, at the time of closing our database, of 27 D2T patients on JAKis therapy, 88.9% had achieved remission (25.9%) or LDA (63.0%), which is not necessarily maintained after a switch to another mechanism of action, especially in highly complex patients. A potential loss of clinical response may often lead to an overuse of symptom-modifying drugs such as NSAIDs (intrinsically associated with increased CV risk (15)) and corticosteroids (associated with increased infectious and CV risk (16, 17)). Moreover, a well-defined link exists between disease activity and the disease-related increase in the risk of developing precisely the same adverse events that should be prevented by discontinuation of JAKis in ineligible patients, including VTE (18), serious infections (19, 20), malignancy (21), and MACEs (22).

In this clinical scenario, it is important to emphasize the disproportion between the limitations proposed by the EMA and what emerges from the real-life experience of registries and claim databases, which did not report an increase associated with the use of JAKis in MACEs (9, 23, 24), VTEs (23, 25), malignancy (23, 24), and serious infections (9, 23) compared to TNFis, even in patients >65 years old. Accordingly, also in our cohort the incidence of adverse events of special interest was very low, even limiting the analysis to the subgroup of patients >65 years old and classified as ineligible according to the EMA pronouncement. Furthermore, a proper definition of a ‘smoking patient’ for the purposes of prescribing JAKis that takes into account the amount and duration of smoking has not yet been conclusively established.

For all these reasons, it may be crucial for clinical practice to interpret the EMA’s indications correctly in a non-overly restrictive sense.

The analysis of the individual factors leading to the definition of ineligibility in our cohort showed as the main cause the increased CV risk (41.8%), which in a large proportion of patients (19.3%) was found to be the only reason for ineligibility to JAKis therapy. Given the relevance of this question, we decided to re-quantify CV risk using a prediction algorithm, as advocated by international recommendations on CV disease prevention (26, 27). Our choice fell on a score validate for RA, the ERS-RA, which in an analysis conducted in an Italian cohort showed a better, although not statistically significant, ability to predict CV risk in patients with RA than other scoring systems such as the Systematic COronary Risk Evaluation (SCORE), the Framingham Risk Score (FRS), the Reynolds Risk Score (RSS) or the American College of Cardiology/American Heart Association score (ACC/AHA 2013) (28, 29). Compared with the use of the inclusion criteria of the ORAL SURVEILLANCE study (8), the ERS-RA enabled to halve the proportion of subjects classified as being at risk, thus reducing the rate of patients ineligible due to increased CV risk alone to 5% and the overall proportion of patients ineligible for JAKis therapy to 46.4%. The only case of MACE observed in our cohort occurred in a patient in whom ERS-RA showed an increased CV risk, confirming the good specificity of the tool.

In contrast to the ORAL SURVEILLANCE study (8), we did not observe an increased incidence of adverse events of special interest in subjects >65 years compared to younger patients. This result is also consistent with what has been reported in the main real-life observational studies (23–25).

The present study certainly has some limitations. The relatively low sample size and the mean follow-up period of approximately 2 years partially limit the ability to intercept uncommon adverse events (such as VTEs) and those that may occur following more prolonged drug exposure (such as MACEs and malignancies). Furthermore, the low number of patients enrolled did not allow a comparative analysis between individual JAKis, which could have been useful to investigate the topic of a potential different selectivity of each drug for members of the JAK family (30). However, it should be considered that in Europe JAKis were licensed in 2017, making the exposure period in our and any other European cohort no longer than 5 years.

On the other hand, our study has the strength of being the first to analyze in detail the impact of the EMA pronouncement on clinical practice and of having been conducted in a single center, which ensured great uniformity and completeness of the data collected. Moreover, in terms of the prevalence of risk factors for adverse events, the baseline characteristics of our cohort were perfectly consistent with those reported by the main observational studies conducted on this aspect (31–33), making our analysis and results plausible.

In conclusion, our analysis shows how the stringent application of the criteria proposed by EMA risks to considerably limit the use of a drug class that has proved to be effective, particularly in D2T RA where treatment options, excluding JAKis, would become very limited. In our experience, the use of ERS-RA seems to be an excellent solution for a proper quantification of CV risk with the aim to define patient ineligibility for JAKi therapy. Obviously, our preliminary results will hopefully have to be confirmed over longer observational periods in prospective studies or larger multicentric registries.

## Data availability statement

The raw data supporting the conclusions of this article will be made available by the authors, without undue reservation.

## Ethics statement

The studies involving humans were approved by Gaetano Pini Institute Ethics Committee. The studies were conducted in accordance with the local legislation and institutional requirements. The participants provided their written informed consent to participate in this study.

## Author contributions

EF and RC contributed to conception and design of the study. GC, SG, RT, FO, GM and MF organized the database. MF performed the statistical analysis. GC wrote the first draft of the manuscript. GC, MB, and MF wrote sections of the manuscript. All authors contributed to manuscript revision, read, and approved the submitted version.
